# Alleviation Effects of *Bifidobacterium animalis* subsp. *lactis* XLTG11 on Dextran Sulfate Sodium-Induced Colitis in Mice

**DOI:** 10.3390/microorganisms9102093

**Published:** 2021-10-03

**Authors:** Nana Wang, Song Wang, Baofeng Xu, Fei Liu, Guicheng Huo, Bailiang Li

**Affiliations:** 1 Key Laboratory of Dairy Science, Ministry of Education, Northeast Agricultural University, Harbin 150030, China; nanawang1017@163.com (N.W.); ws_1984@163.com (S.W.); xubaofeng93@163.com (B.X.); david.as@163.com (F.L.); guichenghuo@126.com (G.H.); 2 Food College, Northeast Agricultural University, Harbin 150030, China

**Keywords:** *Bifidobacterium animalis* subsp. *lactis*, colitis, inflammation, tight junction proteins, gut microbiota

## Abstract

Inflammatory bowel disease (IBD) is a chronic immune-related disease, which can occur through the dysfunction of the immune system caused by the imbalance of gut microbiota. Previous studies have reported the beneficial effects of *Bifidobacterium* on colitis, while the related mechanisms behind these effects have not been fully elucidated. The aim of our study is to investigate the alleviation effect of *Bifidobacterium animalis* subsp. *lactis* XLTG11 *(B. lactis*) on dextran sulfate sodium (DSS)-induced colitis and its potential mechanism. The results showed that *B. lactis* XLTG11 significantly decreased weight loss, disease activity index score, colon shortening, myeloperoxide activity, spleen weight, and colon tissue damage. Additionally, *B. lactis* XLTG11 significantly decreased the levels of pro-inflammatory cytokines and increased the level of anti-inflammatory cytokine. Meanwhile, high doses of *B. lactis* XLTG11 significantly up-regulated the expression of tight junction proteins and inhibited activation of Toll-like receptor 4 (TLR4)/myeloid differentiation factor 88 (MYD88)/nuclear factor-κB (NF-κB) signaling pathway. Furthermore, *B. lactis* XLTG11 increased the gut microbiota diversity and modulated gut microbiota composition caused by DSS. Moreover, Spearman’s correlation analysis also found that several specific gut microbiota were significantly correlated with colitis-related indicators. These results demonstrated that *B. lactis* XLTG11 can alleviate DSS-induced colitis by inhibiting the activation of the TLR4/MYD88/NF-κB signaling pathway, regulating inflammatory cytokines, improving intestinal barrier function, and modulating the gut microbiota.

## 1. Introduction

Inflammatory bowel disease (IBD) is a chronic and recurrent autoimmune disease that includes ulcerative colitis (UC) and Crohn’s disease (CD). Although IBD is a global health burden with the highest incidence in Western countries, the incidence in newly industrialized countries such as Asia, Africa, and South America has shown a rapid upward trend [1]. The specific pathogenesis of IBD remains unclear, accumulating data indicates that genetic susceptibility, abnormal immune response, damaged intestinal barrier, and intestinal microflora imbalances play a vital role in the occurrence and progression of IBD [2,3,4]. The traditional drugs, including aminosalicylates, immunosuppressants, and biological drugs in patients with IBD, pose serious side effects, such as headache, nausea, and infection [2,5,6]. Therefore, it is important to develop effective and harmless alternative strategies to alleviate IBD symptoms.

The gut microbiota plays a crucial role in human energy metabolism and immunity. There is growing evidence that an unfavorable alteration of gut microbiota is associated with the development of human diseases, such as obesity, allergy, IBD, and type 2 diabetes [3,7,8]. The composition of gut microbiota is closely related to the host immune system [9]. Firmicutes and Bacteroidetes are the most abundant in gut microbiota, which is closely related to intestinal health [10]. Compared with healthy individuals, the decrease of Bacteroides and increase of Firmicutes are observed in patients with IBD [11,12,13]. It has been demonstrated that potential pathogenic bacteria (Escherichia coli and Proteobacteria) are increased in patients with UC when compared to healthy subjects [14]. The pathogenic bacteria invade intestinal epithelial cells, stimulate inflammation, damage the integrity of the intestinal epithelial barrier, and triggers intestinal inflammatory responses [15]. Thus, gut microbiota might be a potential and important target for UC therapy.

Probiotics are live microorganisms that colonize the human intestine to exert beneficial effects on the intestinal tract [16]. Particularly, *Lactobacilli* and *Bifidobacteria* are the two main probiotic candidates for intervention in intestinal inflammation [17]. *Bifidobacteria* probiotics play specific roles in reducing the relative abundance and colonization of conditioned pathogens, host-microbial homeostasis, protecting the integrity of the intestinal mucosal barrier, and intestinal inflammation regulation [18,19,20,21]. Moreover, several researchers have reported the beneficial effects of *Bifidobacterium* on colitis. Previous studies found that *Bifidobacterium breve* was effective in alleviating DSS-induced colitis by inhibiting the inflammatory cytokines, enhancing the intestinal epithelial barrier, and regulating the gut microbiota. Din et al. indicated that *Bifidobacterium bifidum* ATCC 29521 alleviated DSS-caused ulcerative colitis by modulating miRNA-associated tight junction proteins and NF-κB regulation and by partially restoring dysbiosis [22]. However, the related mechanisms of *Bifidobacterium* improve colitis have not been fully elucidated.

*Bifidobacterium animalis* subsp. *lactis* XLTG11 was found to have strong acid and bile salts tolerance, high cell adhesion properties, and anti-inflammatory ability in vitro. Therefore, this study aimed to investigate the alleviation effect of *B. lactis* XLTG11 on DSS-induced colitis in mice and its potential mechanism. In the current study, Disease activity index (DAI) score, colon length, MPO activity, spleen index, and histopathological analysis were determined to evaluate the effect of *B. lactis* XLTG11 on DSS-induced colitis. We investigated the inflammatory cytokines, intestinal barrier key gene expression, TLR4/NF-κB signaling pathway, and gut microbiota to assess the potential mechanisms involved. Our results would provide a new insight for probiotics of *B. lactis* XLTG11 to alleviate colitis.

## 2. Materials and Methods

### 2.1. Bacterial Strain and Culture

*Bifidobacterium animalis* subsp. *lactis* XLTG11 was isolated from Healthy children’s intestines in China and preserved at China General Microbiological Culture Collection Center. The strain was anaerobically cultured in modified De Man Rogosa Sharpe (mMRS) medium (Hopebio Company, Qingdao, China, HB0384-5) supplemented with 0.05% L-cysteine hydrochloride under conditions at 37 °C for 18 h and sub-cultured twice prior to the experiment. The Bacteria were obtained by centrifugation (6000× *g* for 10 min at 4 °C) and washed three times with sterile phosphate-buffered saline (PBS). The *B. lactis* XLTG11 was re-suspended at 1 × 10^7^ and 1 × 10^8^ CFU/mL in PBS buffer.

### 2.2. Animals and Experimental Design

Eight-week-old specific pathogen-free (SPF) C57BL/6 male mice were purchased from Beijing Vital River Laboratory Animal Technology Co., Ltd. (Beijing, China). The mice were housed at an ambient condition under 23 ± 2 °C, 50 ± 10% humidity, and 12 h of the light-dark cycle. All animal procedures were performed in accordance with the Guidelines for Care and Use of Laboratory Animals of Northeast Agricultural University and the experiments were approved by the Animal Ethics Committee of Northeast Agricultural University (ethic approval code: NEAUEC2001119). All animals were acclimatized for one week prior to the experiments. The mice were randomly divided into four groups: normal control group (NC), model control group (MC), low doses of *B. lactis* XLTG11 (BL), and high doses of *B. lactis* XLTG11 (BH). During the whole experiment, the BL and BH groups were treated with low doses of *B. lactis* XLTG11 (1 × 10^7^ CFU/d), high doses of *B. lactis* XLTG11 (1 × 10^8^ CFU/d) by oral gavage once daily, respectively. The NC and MC groups were gavaged with 200 μL PBS once daily at the same feeding frequency. From days 15 to 21, all mice except the NC group received 2.5% DSS (Alphabio, Tianjin, China) dissolved in drinking water to induce colitis [23]. During DSS treatment, the bodyweight of all mice was measured every day, and the DAI score was recorded according to bodyweight loss, stool consistency, and gross blood as previously described [2].

At the end of the experiments, the mice were fasted for 12 h, anesthetized with ether, due to the strong anesthetic effect, good safety, and muscle relaxation effect [24,25]. The blood samples of all mice were collected by taking the eyeballs and serum was obtained by centrifuged for 15 min at 1500× *g* at 4 °C and stored at −80 °C. The colon contents were collected under sterile conditions and then stored at −80 °C for gut microbiota analysis. The colon length was measured and washed with ice-cold physiological saline, then resected colon tissue was fixed in 4% paraformaldehyde immediately for histopathological analysis, and the remaining tissues were stored at −80 °C for a real-time quantitative polymerase chain reaction. The spleen was also harvested and weighed, and the spleens index was calculated as follows: spleens index (%) = $\frac{\mathrm{liver}\;\mathrm{weight}}{\mathrm{body}\;\mathrm{weight}}\times 100$.

### 2.3. Histopathological Analysis

The distal colon was fixed in 4% paraformaldehyde for 48 h, embedded in paraffin, and cut into 5 µm sections. These slices were dewaxed with xylene and then stained with hematoxylin and eosin (H&E) for observation. Histological scores were calculated as described previously [26].

### 2.4. Colonic MPO Activity and Serum Proinflammatory Cytokines

The colonic tissues of mice in different treatment groups were weighed and homogenized in the manufacturer’s stocking buffer (1:19), then measured by the myeloperoxide (MPO) test kit (Nanjing Jiancheng Bioengineering Institute, Nanjing, China) according to the manufacturer’s instructions. The levels of interleukin-1β (IL-1β), IL-10, IL-6, and tumor necrosis factor-alpha (TNF-α) in serum were measured with the ELISA kits (Quanzhou Kenuodi Bio-Technology Co., Ltd., Quanzhou, China) according to the manufacturer’s instructions.

### 2.5. Real-Time Quantitative Polymerase Chain Reaction (qRT-PCR)

The relative mRNA levels of tight junction protein genes (claudin-1, occluding and ZO-1) and TLR4 signaling pathway related genes (TLR4, MYD88 and NF-κB) were detected by quantitative real-time polymerase chain reaction (qRT-PCR), and GAPDH gene was used as an internal reference gene. The primers used for qRT-PCR were described in previous studies [27]. Total RNA of the colon was extracted with RNAiso Plus ((Takara Biotechnology, Dalian, China) and quantified by using 2000C Ultra-micro UV spectrophotometer (Thermo Fisher Scientific Inc., Waltham, MA, USA). The mRNA was reverse transcribed into cDNA with Transcriptor First Strand cDNA Synthesis Kit (Roche, Germany, 04897030001). qRT-PCR was carried out according to the instructions on the Bio-Rad CFX96 real-time PCR System (Bio-Rad, Foster City, CA, USA) using Stormstar Sybrgreen qPCR Master Mix kit (DBI Bioscience, Ludwigshafen, Germany, DBI-2143). The expression of related genes was analyzed using the 2^−ΔΔCT^ method.

### 2.6. Gut Microbiota Analysis

The colon microbiota genomic DNA in each group (*n* = 3) was extracted using the E.Z.N.A.^®^ Stool DNA Kit (Omega Bio-Tek, Norcross, GA, USA) according to the manufacturer’s recommendations. The V3-V4 region of the bacterial 16S rDNA was amplified by PCR using the 338F and 806R primers: (5′-ACTCCTACGGGAGGCAGCAG-3′) (forward primer) and (5′- GGACTACHVGGGTWTCTAAT-3′) (reverse primer). The resulting PCR was purified using an AxyPrep DNA gel extraction kit (Axygen Biosciences, Union City, CA, USA), and quantified using Qubit 2.0 Fluorometer (Life Technologies, Carlsbad, CA, USA). Sequencing was performed on the Illumina Miseq platform (Illumina Inc., San Diego, CA, USA) according to standard protocols. The raw data was merged with Flash (V1.2.11) software and filtered by QIIME (V1.9.1) to collect the high-quality clean tags [28,29]. The effective tags were clustered by UCLUST (version 1.2.22) into OTUs of ≥97% similarity [30]. OTUs were analyzed based on the Greengenes database by PyNAST software (Version 1.2) and annotated with taxonomic information at phylum and genus levels [31].

### 2.7. Statistical Analysis

All data were analyzed using SPSS 22.0 software (SPSS Inc., Chicago, IL, USA) and expressed as the mean ± standard deviation (SD). The statistical difference was determined using one-way ANOVA, followed by Duncan’s multiple range test. The relationship between dominant gut microbiota and UC-related symptoms was applied by spearman correlation. *p*-value < 0.05 were considered to be statistically significant.

## 3. Results

### 3.1. Effects of B. lactis XLTG11 on DSS-Induced Colitis Symptoms

**Figure 1:**
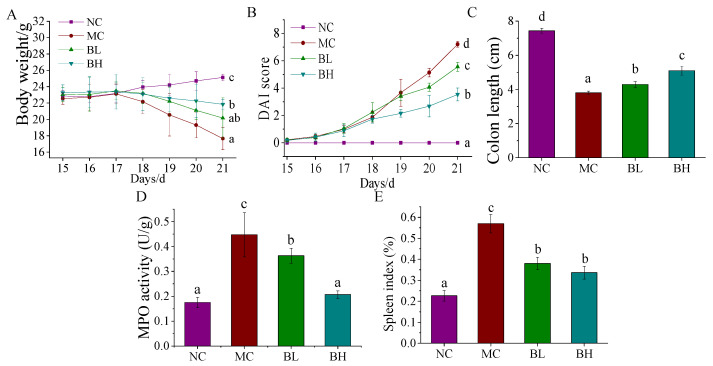
Effects of *B. lactis* XLTG119 on DSS-induced colitis symptoms. (**A**) Bodyweight; (**B**) DAI score; (**C**) Colon length; (**D**) MPO activity; (**E**) spleen index. NC, normal control group; MC, model control group; BL, low-dose *B. lactis* XLTG11; BH, high-dose *B. lactis* XLTG11. All data are expressed as mean ± SD. Different letters indicate statistically significant differences (*p* < 0.05) between the groups.

The body weight during DSS induction was measured in Figure 1A. The NC group showed a steady increase in body weight, but the bodyweight of mice in the MC group showed a significant decrease trend from day 17 until the end of the experiment (*p* < 0.05). Both BL and BH treatment groups reversed these changes to some extent, and the bodyweight of the BH group was significantly higher than that of the MC group (*p* < 0.05). The changes in DAI scores were shown in Figure 1B. The DAI scores of the NC group remained at 0, while the DAI score for the MC group showed a rapid increase trend compared to those of the NC group (*p* < 0.05). The increments in DAI scores for the BL and BH groups were significantly smaller than that of the MC group (*p* < 0.05). The colon length of the mice in four groups was shown in Figure 1C. Compared with the NC group, the colon length showed a significant reduction in the MC group (*p* < 0.05). After the intervention, the symptoms of colon shortening caused by DSS appeared mitigated (*p* < 0.05). As shown in Figure 1D, compared with the NC group, the MPO activity of mice in the MC group was significantly increased (*p* < 0.05), but compared with the MC group, the MPO activity was significantly decreased in a dose-dependent manner in the BL group and BH group (*p* < 0.05), and there was no significant difference between NC group and BH group (*p* < 0.05). The spleen indexes in each treatment group were shown in Figure 1E. We observe that the mice in the MC group were substantially higher compared with the NC group (*p* < 0.05), but both doses of *B. lactis* XLTG11 administrations reversed these changes, and the trend in the BH group was more similar to the NC group. These results indicated that *B. lactis* XLTG11 effectively relieved DSS-induced colitis symptoms.

### 3.2. Effects of B. lactis XLTG11 on Colon Histopathological Analysis

**Figure 2:**
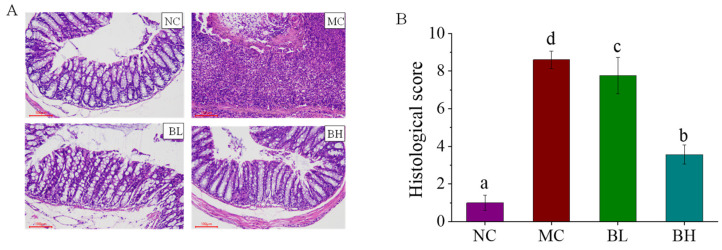
Effects of *B. lactis* XLTG11 on colon histopathological analysis. (**A**) Histological images; (**B**) Histological score. NC, normal control group; MC, model control group; BL, low-dose *B. lactis* XLTG11; BH, high-dose *B. lactis* XLTG11. All data are expressed as mean ± SD. Different letters indicate statistically significant differences (*p* < 0.05) between the groups.

The Histological changes in each treatment group were shown in Figure 2A. The colon tissue of the NC group showed complete goblet cells and intact epithelial tissue, but the crypt structure and goblet cells of the colon tissue of the mice in the MC group were disappeared, and the inflammatory cells infiltrated. After being treated with *B. lactis* XLTG11, the colonic tissues in the BL group and BH group showed improved structural damage and reduced inflammatory cell infiltration. In addition, compared with the MC group, both doses of *B. lactis* XLTG11 administrations dramatically reduced histology scores caused by DSS (Figure 2B; *p* < 0.05). In particular, the BH group showed a higher ability to reduce the pathological score relative to the BL group, which was closer to the NC group. These findings demonstrated that a high-dose *B. lactis* XLTG11 significantly improved tissue morphological changes induced by DSS and reduced histology scores.

### 3.3. Effects of B. lactis XLTG11 on Inflammatory Cytokines

**Figure 3:**
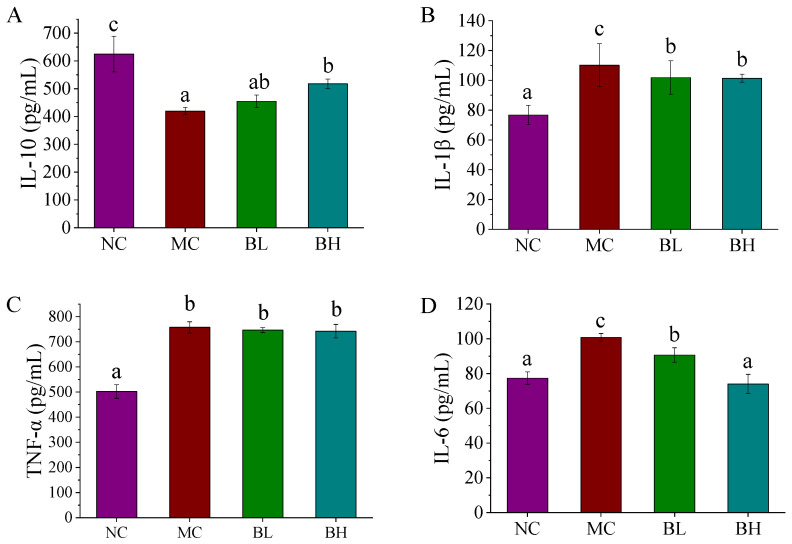
Effects of *B. lactis* XLTG11 on inflammatory cytokines. (**A**) IL-10; (**B**) IL-1β; (**C**) TNF-α and (**D**) IL-6. NC, normal control group; MC, model control group; BL, low-dose *B. lactis* XLTG11; BH, high-dose *B. lactis* XLTG11. All data are expressed as mean ± SD. Different letters indicate statistically significant differences (*p* < 0.05) between the groups.

The levels of inflammatory cytokines in the serum of mice in all groups were shown in Figure 3. The levels of pro-inflammatory cytokines (including IL-1β, TNF-α, and IL-6) significantly increased in the mice in the MC group than that in the NC group. While the levels of IL-1β and IL-6 in both doses of *B. lactis* XLTG11 administrations were lower than that of the MC group (*p* < 0.05). Especially, compared with the NC group, there was no significant difference in the level of IL-6 between the high-dose *B. lactis* XLTG11 group and the NC group (*p* > 0.05). The serum IL-10 levels in the MC group were significantly lower than that of the NC group (*p* < 0.05). Conversely, the levels of IL-10 were significantly reversed by high-dose *B. lactis* XLTG11 interventions (*p* < 0.05), but these changes were not significant in the BL and MC group (*p* > 0.05). The abovementioned data showed that high-dose *B. lactis* XLTG11 administration could effectively inhibit inflammation symptoms caused by DSS relative to the BL group.

### 3.4. Effects of B. lactis XLTG11 on Claudin-1, Occludin, and ZO-1 mRNA Expression

**Figure 4:**
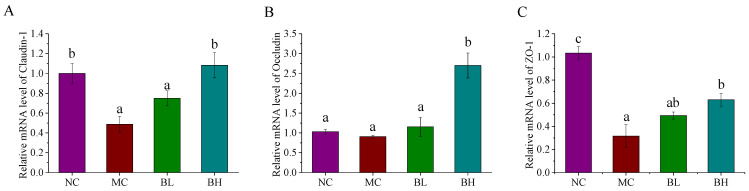
Effects of *B. lactis* XLTG11 on claudin-1 (**A**), occluding (**B**), and ZO-1 (**C**) mRNA expression in colon tissues were detected by qRT-PCR. NC, normal control group; MC, model control group; BL, low-dose *B. lactis* XLTG11; BH, high-dose *B. lactis* XLTG11. All data are expressed as mean ± SD. Different letters indicate statistically significant differences (*p* < 0.05) between the groups.

The tight junction proteins (claudin-1, occludin, and ZO-1) in the colon were shown in Figure 4. Compared with the NC group, the expression levels of claudin-1 and ZO-1 in the MC group were significantly decreased (*p* < 0.05), implying that the epithelial integrity has been impaired. Compared with the MC group, the expression levels of claudin-1 and ZO-1 were significantly reversed by high-dose *B. lactis* XLTG11 supplementation (*p* < 0.05), but these changes were not significant in the low-dose *B. lactis* XLTG11 and MC group (*p* > 0.05). In addition, the occludin mRNA expression levels in the MC group were no significant differences when compared to the NC group (*p* > 0.05). Noticeably, after high-dose *B. lactis* XLTG11 supplementation, the expression levels of occludin were pronouncedly increased than that in the MC group. These results showed that the high dose of *B. lactis* XLTG11 supplementations could ameliorate the intestinal barrier function induced by DSS.

### 3.5. Effects of B. lactis XLTG11 on the TLR4/MYD88/NF-ĸB Signaling Pathway

**Figure 5:**
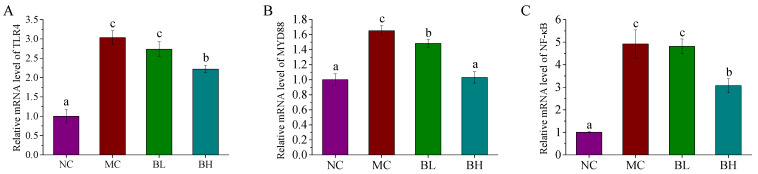
Effects of *B. lactis* XLTG11 on TLR4 (**A**), MYD88 (**B**), and NF-ĸB (**C**) mRNA expression levels in colon tissues were detected by qRT-PCR. NC, normal control group; MC, model control group; BL, low-dose *B. lactis* XLTG11; BH, high-dose *B. lactis* XLTG11. All data are expressed as mean ± SD. Different letters indicate statistically significant differences (*p* < 0.05) between the groups.

To investigate whether the TLR4/MYD88/NF-ĸB signaling pathway plays a significant role in the regulation of *B. lactis* XLTG11 on anti-inflammatory mechanisms, the mRNA expression levels of its related genes were measured. As shown in Figure 5, DSS administration remarkably increased the mRNA expression levels of TLR4, MYD88 and NF-ĸB compared to those of the NC group (*p* < 0.05). Conversely, the TLR4 and NF-ĸB gene expression were observably down-regulated in the BH groups (*p* < 0.05), but there were no significant differences between the BL and MC groups (*p* > 0.05). Furthermore, both doses of *B. lactis* XLTG11 dramatically down-regulated the mRNA expression of MYD88 when compared with the MC group (*p* < 0.05). Especially, treatment with the high-dose *B. lactis* XLTG11 was similar to that of the NC group (*p* > 0.05). These findings indicated that a high-dose *B. lactis* XLTG11 exerted its anti-inflammatory function by inhibiting TLR4/MYD88/NF-ĸB activation. 

### 3.6. Effects of B. lactis XLTG11 on Structure and Composition of Gut Microbiota 

**Figure 6:**
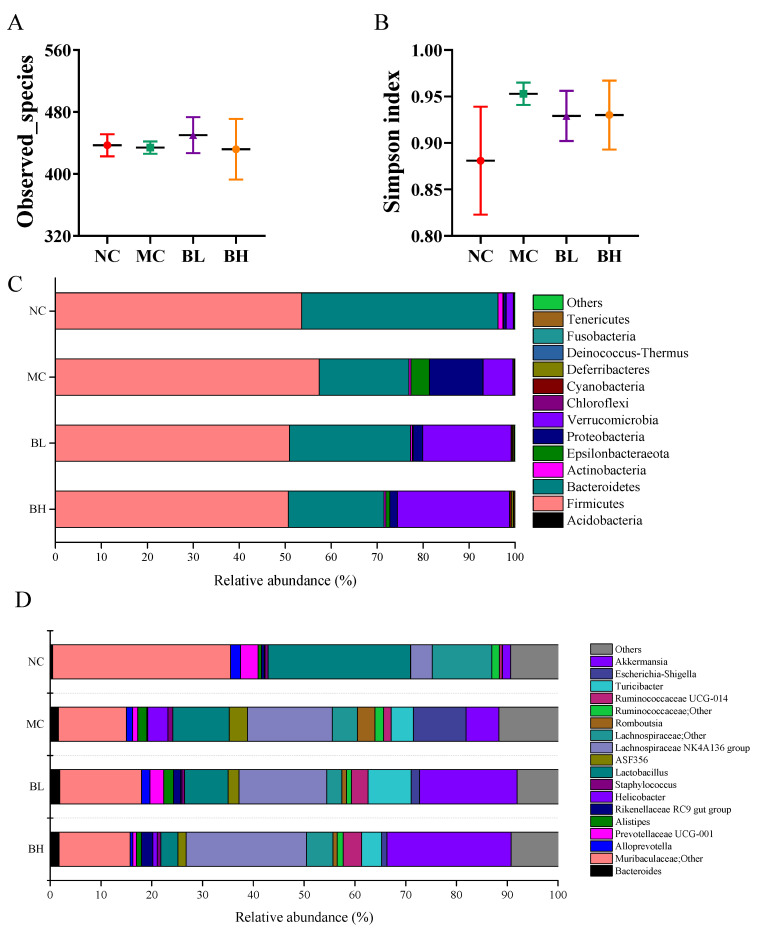
Effects of *B. lactis* XLTG11 on structure and composition of gut microbiota. (**A**) Observed_species; (**B**) Simpson index; (**C**) Gut microbiota composition at the phylum level; (**D**) Gut microbiota composition at the genus level. NC, normal control group; MC, model control group; BL, low-dose *B. lactis* XLTG11; BH, high-dose *B. lactis* XLTG11.

Three out of the 8 mice were randomly selected as part of an exploratory pilot analysis. The gut microbiota of the colonic contents among different groups were analyzed by sequencing the V3-V4 hypervariable region of the 16S rDNA gene. The observed_species of the mice in the MC group was not significantly different from that in the NC group (Figure 6A), and Simpson index of the mice in the MC group was higher than that in the NC group (Figure 6B), implying that DSS intake resulted in changes in the microbial diversity. Whereas, *B. lactis* XLTG11 administration reversed these changes to a certain extent.

At the phylum level, compared with the NC group, reduced relative abundances of Bacteroidetes and increased relative abundances of Firmicutes, Epsilonbacteraeota and Proteobacteria were observed in the MC group (Figure 6C and Figure S1). Therefore, the Firmicutes/Bacteroidetes (F/B) ratio was higher than the other groups. However, the F/B ratio, Epsilonbacteraeota and Proteobacteria were reduced in the BL and BH group to similar levels as those in the NC group. At the genus levels (Figure 6D and Figure S2), the relative abundances of *Helicobacter*, *Escherichia-Shigella*, *Romboutsia*, and *Staphylococcus* was higher in the MC group than that in the NC group (*p* < 0.05). The relative abundance of *Helicobacter* was significantly decreased in BL group (*p* < 0.05). Furthermore, the relative abundance of *Muribaculaceae*; Other was significantly decreased and *Lachnospiraceae NK4A136 group* was significantly increased in the MC group compared to the NC group (*p* < 0.05). *B. lactis* XLTG11 supplementation increased the relative abundance of *Muribaculaceae;Other*, *Ruminococcaceae UCG-014*, *Lachnospiraceae NK4A136 group* and *Akkermansia* to some extent, although the changes are not statistically different. Compared with the NC group, the relative abundances of *Alistipes* and *Turicibacter* in the MC group were increased (*p* > 0.05). Whereas the relative abundances of *Alistipes* was decreased only in the high-dose group compared to the MC group (*p* > 0.05). Interestingly, both low-dose and high-dose *B. lactis* XLTG11 administration reduced the proportion of *Lactobacillus* compared to the MC group (*p* > 0.05).

### 3.7. Correlation Analysis between UC-Related Symptoms, Related Gene Expression and Dominant Gut Microbiota 

**Figure 7:**
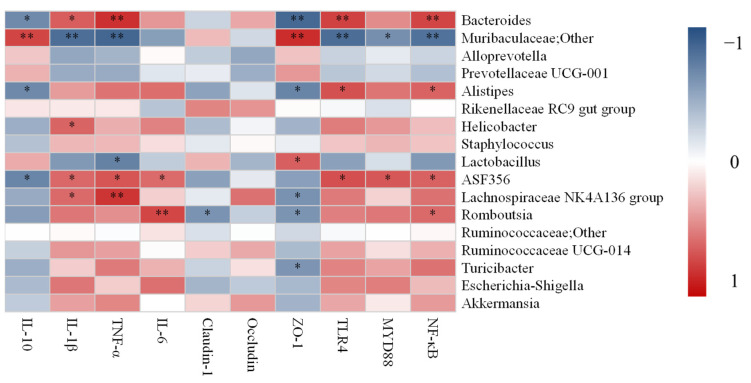
Correlation analysis between UC-related symptoms, related gene expression, and dominant gut microbiota by Spearman. * and ** indicate the associations significant (*p* < 0.05 and *p* < 0.01, respectively).

To determine the role of gut microbiota in alleviating inflammation biomarkers, the correlation between UC-related symptoms, related gene expression, and dominant gut microbiota at the genus levels was analyzed (Figure 7). The relative abundance of *Bacteroides* was significantly positively correlated with IL-1β, TNF-α, TLR4, and NF-κB, while was significantly negatively correlated with IL-10 and ZO-1. Unclassified *Muribaculaceae* genus and *Alloprevotella* were positively associated with IL-10 and ZO-1, while was negatively associated with IL-1β, TNF-α, TLR4, MYD88, and NF-κB. However, the *Alistipes* was positively associated with TLR4 and NF-κB but was significantly negatively correlated with IL-10 and ZO-1. The relative abundance of *Helicobacter* was positively associated with IL-1β. The relative abundances of *Lactobacillus* were significantly negatively correlated with TNF-α, while was significantly positively correlated with ZO-1. The relative abundances of *ASF356* were positively correlated with the pro-inflammation cytokines (IL-1β, TNF-α, and IL-6) and TLR4/MYD88/NF-ĸB signaling pathway, but was negatively correlated with IL-10. The relative abundances of the *Lachnospiraceae NK4A136 group* were positively associated with IL-1β and TNF-α, while was negatively associated with ZO-1. The *Romboutsia* was positively correlated with IL-6 and NF-ĸB but was negatively correlated with Claudin-1 and ZO-1. The relative abundances of *Turicibacter* significantly negatively correlated with ZO-1. The *Escherichia-Shigella* was positively associated with pro-inflammation cytokines (IL-1β, TNF-α, and IL-6) and TLR4/MYD88/NF-ĸB signaling pathway, while was negatively associated with tight junction proteins (claudin-1, occludin, and ZO-1) and IL-10. The *Akkermansia* was positively correlated with the pro-inflammation cytokines (IL-1β and TNF-α), Claudin-1, Occludin, and TLR4/MYD88/NF-ĸB signaling pathway, but was negatively correlated with IL-10 and ZO-1.

## 4. Discussion

The prevalence of IBD is increasing globally, placing a huge health burden on families and society. At present, traditional drugs can cause side effects, so it is imperative to explore natural and harmless therapeutic agents. A large number of in vitro and in vivo studies have shown that some *Bifidobacteria* can effectively adjust intestinal dysfunction [23,32]. In this study, hence, we evaluated the alleviation effect of *B. lactis* XLTG11 on DSS-induced colitis in mice.

DSS model is a typical and standard experimental model of ulcerative colitis [33]. Previous studies reported that DSS treatment exhibited a number of colitis symptoms, such as weight loss, higher DAI score, colon shortening, and increased spleen weight [34]. MPO is an enzyme located in neutrophil granules, which reflects the degree of neutrophil infiltration in colon tissue, and its activity is positively correlated with the degree of inflammation in colon tissue [35]. DSS will damage the intestinal epithelial barrier, infiltrating inflammatory cells, reduce or even disappear goblet cells. In this experiment, it was found that *B. lactis* XLTG11 supplementations could relieve weight loss, decrease DAI score, increase colon length, decrease MPO activity and spleen weight induced by DSS in a dose-dependent manner. After the intervention of *B. lactis* XLTG11, the histopathological score was significantly decreased and the histopathological status of the colon was improved.

The pathogenesis of IBD is related to the dysregulation and hyper-activated intestinal mucosal immune response [35]. It has been reported that the levels of pro-inflammatory cytokines (including IL-1β, TNF-α, and IL-6) were up-regulated, while the levels of anti-inflammatory cytokine IL-10 down-regulated accordingly in the DSS-induced mice [36]. High levels of IL-1β are closely related to the severity of intestinal inflammation and disease activity, and excessive IL-1β can increase intestinal permeability, promote the activation of dendritic cells and macrophages, and play a key role in the pathogenesis of intestinal inflammation [37,38]. TNF-α and IL-6 are considered to be important mediators of the inflammatory response in patients with IBD. Studies have found that TNF-α increases the expression of epithelial myosin light chain kinase, leading to increased intestinal permeability in patients with intestinal inflammation [39]. IL-6 plays an important role in the intestinal epithelial barrier and can regulate the tight junctions of the intestinal epithelium by activating the claudin-2 gene [40]. Anti-IL-6 and anti-TNF-α have become therapeutic targets for the abnormal inflammatory response in patients with IBD. IL-10 is a major anti-inflammatory cytokine and plays a crucial role in maintaining gastrointestinal homeostasis [41]. A study showed that the probiotic mixture can significantly increase the expression of IL-10 in DSS-induced mice, which is essential for reducing intestinal inflammation [42]. In the current study, DSS treatment significantly increased the concentrations of IL-1β, TNF-α, and IL-6, but decreased IL-10 in the MC group compared to that of the NC group. While both dose *B. lactis* XLTG11 interventions reversed these changes to some extent, and high-dose *B. lactis* XLTG11 interventions were particularly effective in regulating cytokines.

The intestinal barrier plays an essential role in the maintenance of intestinal homeostasis and the development of intestinal inflammation [43]. Tight junctions mainly include transmembrane proteins (Occludin and Claudins) and accessory proteins (ZO-1 and ZO-2), which are the main components of the intestinal mucosal barrier and affect the permeability and integrity of the intestinal mucosa [44]. The lack of tight junctions will increase the permeability of the intestinal barrier, resulting in the invasion of bacteria and potentially harmful antigens, and then triggering and promoting the occurrence of intestinal inflammation [45]. Here, we found that DSS administration significantly down-regulated the mRNA expression of claudin-1 and ZO-1 compared to the NC group, indicating that permeability of the intestinal epithelium may be increased. As expected, BH supplements were the most effective in upregulating claudin-1, occludin, and ZO-1 expression in DSS-induced mice followed by the BL treatment. In addition, the previous study has also indicated that *B. longum* CCM 7952 could increase the expression of ZO-1 and occludin in the intestinal epithelium and reduce colon permeability in DSS-treated mice, which is consistent with our results [46].

To further investigate the potential mechanisms of *B. lactis* XLTG11 alleviating the symptoms of DSS-induced colitis in mice, we measured the expression of immune pathway-related genes in colon tissue. Numerous evidence disclosed that the activation of the TLR4/MYD88/NF-κB signaling pathway is associated with the pathogenesis of colitis [47,48]. TLR4-mediated signaling pathway performs intracellular signal transduction by recognizing the ligand and then combining with its main adaptive molecule MYD88, which ultimately leads to the activation of the key transcription factor NF-κB and the secretion of TNF-α, IL-6, and other cytokines by effector cells, thus destroying intestinal immune homeostasis, and eventually leading to UC [49,50,51]. 

TLR4 is the first discovered transmembrane receptor in the TLR family, which has the functions of regulating immune response and pro-inflammatory [52]. MyD88 signal is an important adaptor protein in the process of TLR4 signal transduction, and it is also the upstream signal molecule of the NF-ĸB signaling pathway, which in turn triggers the signal cascade, resulting in the activation of downstream NF-κB [53]. NF-κB is a marker of intestinal mucosal infection, and the level of NF-κB in colon tissue can reflect the severity of UC disease [54]. Studies have also shown that NF-κB is over-activated in the process of UC, leading to the secretion of pro-inflammatory cytokines, and the accumulation of these inflammatory cytokines is considered to be an important factor in the pathogenesis of colitis [55,56]. In this study, the mRNA levels of TLR4, MyD88, and NF-ĸB were remarkably increased in the MC group compared with the NC group. In particular, these changes were significantly decreased by the treatment of BL and BH. Especially, treatment with the high-dose B. *lactis* XLTG11 was closer to that of the NC group, which was similar to the research of Xu et al. [57].

Accumulating studies reported that dysbiosis of the gut microbiota was observed in IBD [58]. The alteration of gut microbiota in patients with IBD is characterized by the reduction of good bacteria and the overgrowth of pathogenic bacteria [59]. Chen et al. demonstrated that *B. breve* could alleviate DSS-induced colitis by regulating the gut microbiota. In the current study, DSS administration increased the Simpson index compared to that in the NC group, which is consistent with the results of sun et al. [60]. However, *B. lactis* XLTG11 supplementation reversed this trend. At the phylum level, the relative abundance of Firmicutes and Proteobacteria was increased, and the relative abundance of Bacteroidetes was decreased in the MC group compared with the NC group, which is consistent with a previous study [61]. In addition, the increase in the abundance of Proteobacteria is regarded as a microbial signature of gut microbiota imbalance [62]. *B. lactis* XLTG11 can suppress the increase of Proteobacteria in DSS-treated mice in a dose-dependent manner, and regulate the dysbiosis of gut microbiota to a certain extent.

At the genus level, the high and low dose groups in mice showed an increase in genera containing probiotics, such as *Muribaculaceae; Other, Akkermansia*, *Ruminococcaceae UCG-014, and Lachnospiraceae NK4A136 group*. Studies have found that *Muribaculaceae; Other* has potential benefits in alleviating inflammation, suppressing harmful bacteria, and/or promoting anti-cancer immunity [63]. Our study also demonstrated that *Muribaculaceae; Other* was positively associated with IL-10 and ZO-1, while was negatively associated with IL-1β, TNF-α, TLR4, MYD88, and NF-κB. *Akkermansia* is associated with intestinal immunity and plays an important role in intestinal homeostasis [64]. It has been pointed out that *Akkermansia* was reduced in patients with IBD, indicating that it may have potential anti-inflammatory effects [65], which was consistent with our study. *Ruminococcaceae UCG-014* is an SCFA producer with an anti-inflammatory effect, which can protect the intestinal barrier and ameliorate colitis [66]. Recent studies found that probiotics and prebiotics administration increased the levels of the *Lachnospiraceae NK4A136 group* caused by DSS, which plays a critical role in relieving colitis [67]. 

In contrast, an opposite trend was observed for some pathogenic genera such as *Helicobacter*, *Escherichia-Shigella*, *Turicibacter*, and *Alistipes*, in which relative abundance was decreased when compared with the MC group. *Helicobacter* and *Escherichia-Shigella* are gram-negative bacteria that can damage the immune system and aggravate intestinal infections, which are usually related to the pathogenesis of UC [68]. Also, Chen et al found that the relative abundance of *Escherichia-Shigella* in DSS-colitis mice was significantly increased, while the administration of Baitouweng decoction could effectively decrease its relative abundance, which was consistent with our research [69]. Bosshard et al. confirmed previously that *Turicibacter* can affect the immune and invasion ability of mice, and cause subclinical infections in piglets [70]. *Alistipes* can worsen recurrent abdominal pain and are closely associated with intestinal inflammation [71]. A previous study found that *Alloprevotella* was increased in DSS induced mice model, which was different from our study [72]. While the role of *Alloprevotella* in digestive diseases remains unclear as it is currently not reported frequently in these diseases. *Lactobacillus* is a probiotic that regulates the gut microbiota, but the proportion of *Lactobacillus* was decreased in the treatment of the BL and BH group. Yan et al. also found that *Lactobacillus acidophilus* administration reduced the relative abundance of *Lactobacillus* in type 2 diabetes mice [73]. Additionally, the correlation analysis in our results also found that *Helicobacter*, *Escherichia-Shigella*, *Turicibacter*, *Alistipes* were positively correlated with pro-inflammation cytokines (IL-1β, TNF-α, and IL-6), TLR4, MYD88, and NF-κB, while were negatively correlated with tight junction proteins (claudin-1, occludin, and ZO-1) and IL-10. Therefore, our results demonstrated that *B. lactis* XLTG11 supplementation could effectively suppress DSS-induced colitis in mice by increasing the proportion of beneficial bacteria and decreasing the relative abundance of some pathogenic genera.

## 5. Conclusions

This study showed that *B. lactis* XLTG11 could alleviate DSS-induced colitis in mice including regulating inflammatory cytokines, protecting intestinal barrier function, inhibiting TLR4/MYD88/NF-κB activation, and modulating the specific gut microbiota. Our research will offer a theoretical basis and future investigations for probiotics to alleviate colitis. 

## Data Availability

Raw sequence datasets from 16S ribosomal RNA gene sequencing are available in the SRA database under accession number RJNA766158.

## Supplementary Materials

The following are available online at https://www.mdpi.com/article/10.3390/microorganisms9102093/s1, Figure S1. Gut microbiota composition at the phylum level; Figure S2. Gut microbiota composition at the genus level.
