# Supplementary material for: Alleviation Effects of Bifidobacterium animalis subsp. lactis XLTG11 on Dextran Sulfate Sodium-Induced Colitis in Mice
Source: Microorganisms. 2021 Oct 3;9(10):2093. doi: 10.3390/microorganisms9102093 (PMC8539219; doi:10.3390/microorganisms9102093)
Supplement: Supplementary file 1 [file microorganisms-09-02093-s001.zip › microorganisms-1341411-SI.pdf]

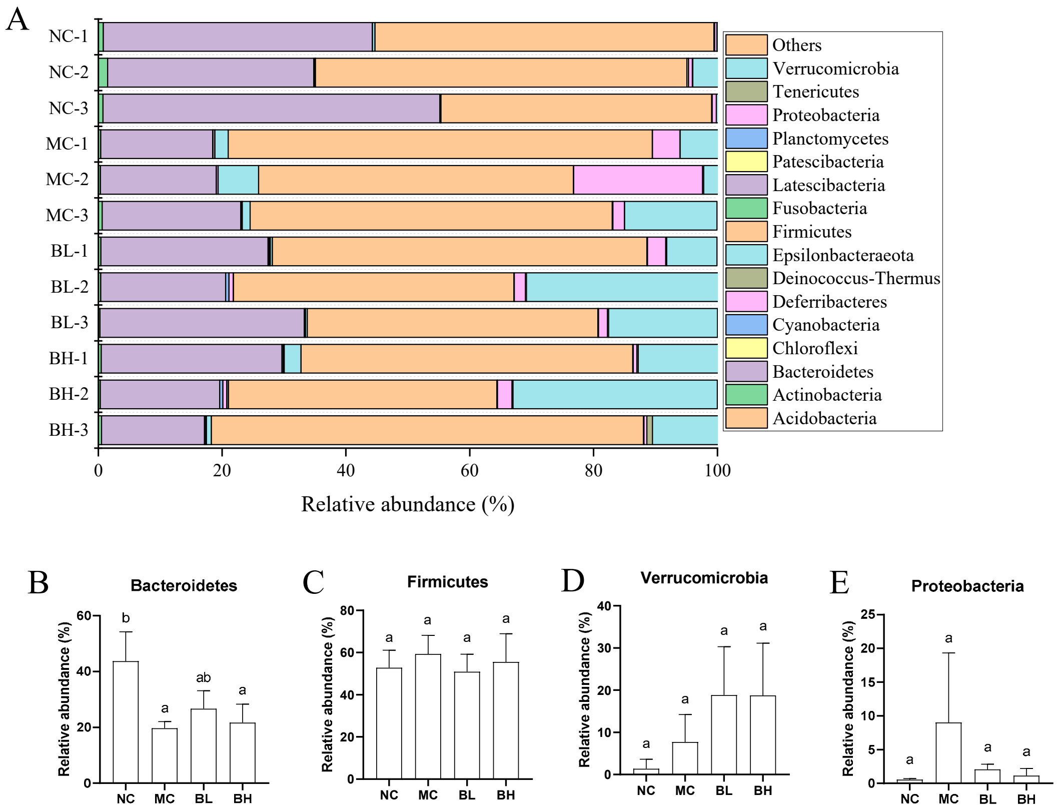

Figure S1. Gut microbiota composition at the phylum level. (**A**) Bar plot of gut microbiota composition at the phylum level; (**B**) Relative abundance of Bacteroidetes across each grouped microbiomes; (**C**) Relative abundance of Firmicutes across each grouped microbiomes; (**D**) Relative abundance of Verrucomicrobia across each grouped microbiomes; (**E**) Relative abundance of Proteobacteria across each grouped microbiome. NC, normal control group; MC, model control group; BL, low-dose *B. lactis* XLTG11; BH, high-dose *B. lactis* XLTG11. All data are expressed as mean ± SD. Different letters indicate statistically significant differences between the groups ($p < 0.05$).

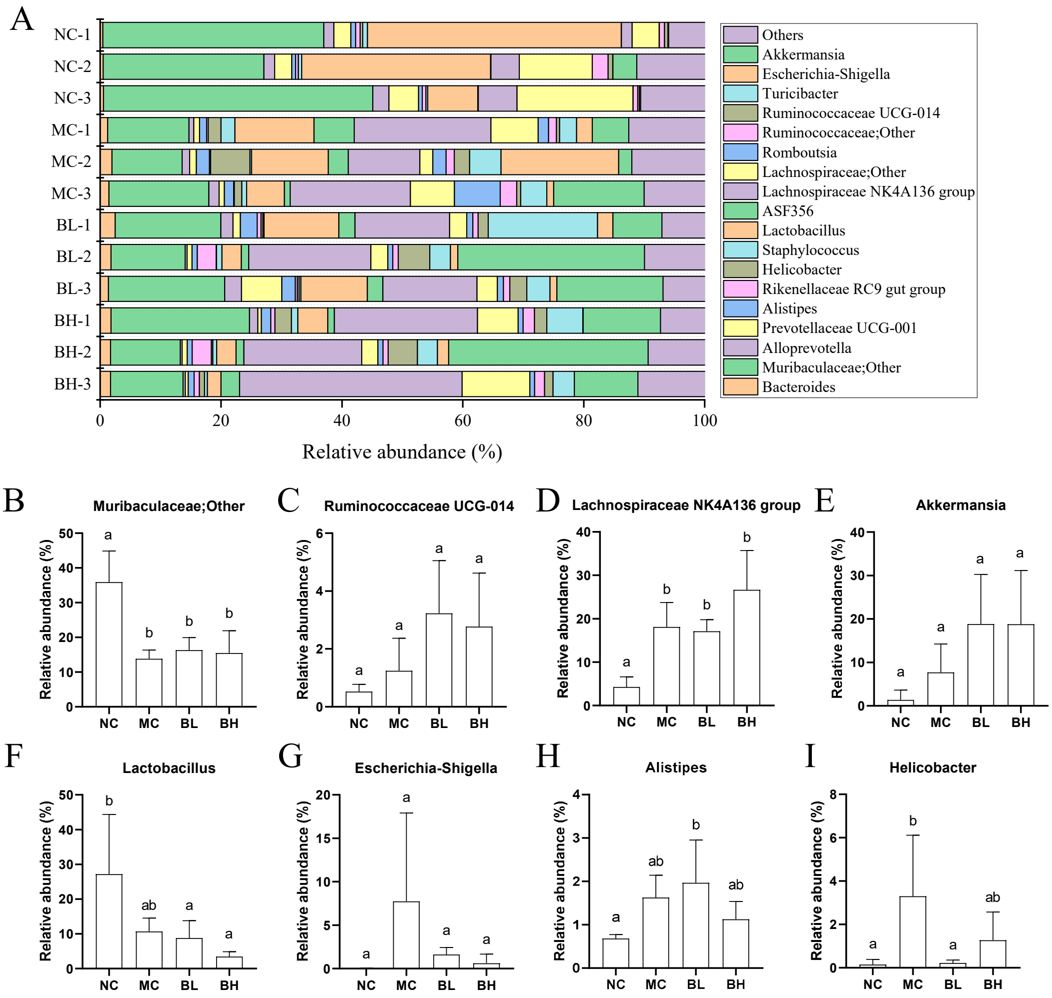

Figure S2. Gut microbiota composition at the genus level. (**A**) Bar plot of gut microbiota composition at the genus level; (**B**) Relative abundance of *Muribaculaceae;Other* across each grouped microbiomes; (**C**) Relative abundance of *Ruminococcaceae UCG-014* across each grouped microbiomes; (**D**) Relative abundance of *Lachnospiraceae NK4A136 group* across each grouped microbiomes; (**E**) Relative abundance of *Akkermansia* across each grouped microbiomes; (**F**) Relative abundance of *Lactobacillus* across each grouped microbiomes; (**G**) Relative abundance of *Escherichia-Shigella* across each grouped microbiomes; (**H**) Relative abundance of *Alistipes* across each grouped microbiomes; (**I**) Relative abundance of *Helicobacter* across each grouped microbiomes. NC, normal control group; MC, model control group; BL, low-dose *B. lactis* XLTG11; BH, high-dose *B. lactis* XLTG11. All data are expressed as mean ± SD. Different letters indicate statistically significant differences between the groups ($p < 0.05$).
